# Empty mesoporous silica particles significantly delay disease progression and extend survival in a mouse model of ALS

**DOI:** 10.1038/s41598-020-77578-x

**Published:** 2020-11-26

**Authors:** Marcel F. Leyton-Jaimes, Patrik Ivert, Jan Hoeber, Yilin Han, Adam Feiler, Chunfang Zhou, Stanislava Pankratova, Varda Shoshan-Barmatz, Adrian Israelson, Elena N. Kozlova

**Affiliations:** 1 Department of Physiology and Cell Biology, Zlotowski Center for Neuroscience, Faculty of Health Sciences, Ben-Gurion University of the Negev, P.O.B. 653, 84105 Beer Sheva, Israel; 2 Department of Neuroscience, Regenerative Neurobiology, Uppsala University Biomedical Center, Box 593, 751 24 Uppsala, Sweden; 3 Nanologica AB, Forskargatan 20G, 151 36 Södertälje, Sweden; 4 Chemistry Department, KTH, Royal Institute of Technology, 100 44 Stockholm, Sweden; 5 Laboratory of Neural Plasticity, Department of Neuroscience, University of Copenhagen, 2200 Copenhagen, Denmark; 6 Comparative Pediatrics and Nutrition, Department of Veterinary and Animal Sciences, University of Copenhagen, 1870 Frederiksberg C, Denmark; 7 Department of Life Sciences, The National Institute for Biotechnology in the Negev Ltd, Ben-Gurion University of the Negev, 84105 Beer Sheva, Israel; 8 Department of Immunology, Genetics and Pathology, Science for Life Laboratory, Uppsala University, Box 815, 751 08 Uppsala, Sweden

**Keywords:** Biotechnology, Neuroscience, Stem cells, Diseases, Neurology, Nanoscience and technology

## Abstract

Amyotrophic lateral sclerosis (ALS) is a devastating incurable neurological disorder characterized by motor neuron (MN) death and muscle dysfunction leading to mean survival time after diagnosis of only 2–5 years. A potential ALS treatment is to delay the loss of MNs and disease progression by the delivery of trophic factors. Previously, we demonstrated that implanted mesoporous silica nanoparticles (MSPs) loaded with trophic factor peptide mimetics support survival and induce differentiation of co-implanted embryonic stem cell (ESC)-derived MNs. Here, we investigate whether MSP loaded with peptide mimetics of ciliary neurotrophic factor (Cintrofin), glial-derived neurotrophic factor (Gliafin), and vascular endothelial growth factor (Vefin1) injected into the cervical spinal cord of mutant SOD1 mice affect disease progression and extend survival. We also transplanted boundary cap neural crest stem cells (bNCSCs) which have been shown previously to have a positive effect on MN survival in vitro and in vivo. We show that mimetic-loaded MSPs and bNCSCs significantly delay disease progression and increase survival of mutant SOD1 mice, and also that empty particles significantly improve the condition of ALS mice. Our results suggest that intraspinal delivery of MSPs is a potential therapeutic approach for the treatment of ALS.

## Introduction

Amyotrophic lateral sclerosis (ALS) is a lethal disease characterized by progressive degeneration of motor neurons (MNs) in the brain and spinal cord that leads to paralysis throughout the whole body. The etiology and mechanisms of ALS are unknown, but emerging evidence indicates that the disease is the result of complex dysfunctional cellular interactions between MNs, astrocytes, and microglia leading to glutamate excitotoxicity, oxidative stress, protein misfolding/aggregation, mitochondrial failure, perturbation of RNA processing, and neuroinflammation^1^. Currently, the approved treatments for ALS, Riluzole and Edaravone, offer only a moderate extension of the patient’s life expectancy. Thus, improved treatment strategies are urgently needed.

A small proportion of ALS patients (5–10%) have a familial form, and of those cases, 20% have a mutation in the gene encoding Cu^2+^/Zn^2+^-superoxide dismutase (SOD1). Similar mutations are found in about 2% of sporadic ALS patients^2^. SOD1 transgenic animals that express human mutant SOD1 develop progressive motor neuron disease and represent a very good model for the human disease^3–8^. Since familial and sporadic patients of ALS present similar clinical symptoms, these models are widely accepted to study the disease pathogenesis. It is today well established that mutant SOD1 proteins cause a selective degeneration of MNs by acquisition of different toxic properties but not via a loss of function mechanism^3–6^. Moreover, although this is still controversial^9–11^, misfolded SOD1 was proposed to occur not only in familial ALS cases with SOD1 mutations, but also in sporadic ALS patients^12–17^, strengthening the translational relevance of studying ALS with SOD1 mutant models such as the SOD1^G93A^ mouse.

Neurotrophic factors, including e.g., ciliary neurotrophic factor (CNTF), glial-derived neurotrophic factor (GDNF), and vascular endothelial growth factor (VEGF), have shown beneficial effects on disease progression in animal models of ALS^18^. These factors have been delivered as protein solutions via viral vectors or over-expressing stem cells. Mesoporous silica nanoparticles (MSPs) are attractive as a highly robust and tunable delivery platform^19,20^. We previously showed that MSPs loaded with trophic factor peptide mimetics induce differentiation of mouse embryonic stem cells (ESC) to MNs in vitro^21^, increase survival of transplanted ESC and guide their differentiation towards MNs in vivo^22^, and support sensory axon regeneration in the spinal cord after dorsal root avulsion^23^.

Transplantation of neural stem cells has shown beneficial effects on disease progression and life span in animal models of ALS, presumably through multiple mechanisms that include trophic support and alleviation of the neuroinflammatory environment^24,25^. Clinical trials with neural stem cells indicate that this intervention is well tolerated and safe, but the symptomatic benefits have so far been, at best, modest^26,27^. We previously showed that neural crest stem cells from the boundary cap (bNCSCs), a transient structure during early development at the entry/exit points of spinal nerve roots, have extraordinary protective effects in vitro and in vivo on insulin producing beta-cells^28–31^, excitotoxically challenged MNs in spinal cord slices^32^, and murine ESC-derived SOD1^G93A^ MNs in vitro and in vivo^33^.

Here, we have performed a comparative study addressing how intraspinal implantation of MSPs loaded with a cocktail of neurotrophic factor mimetics Cintrofin, Gliafin, and Vefin1, unloaded MSP, and bNCSCs affect disease progression in the SOD1^G93A^ mouse model of ALS.

## Results

### Characterization of MSP

**Figure 1:**
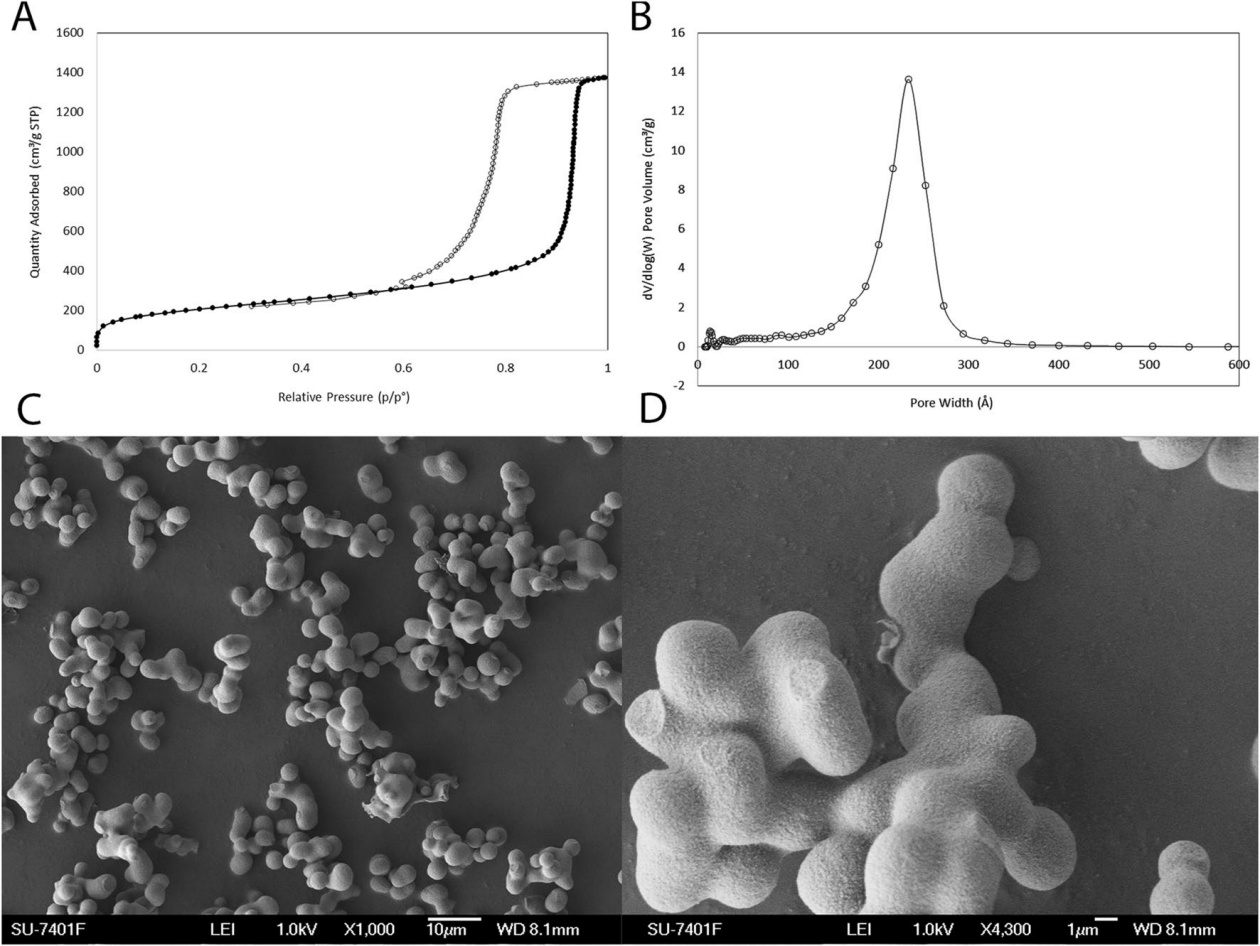
Synthesis and characterization of MSPs. Surface area (**A**) and porosity (**B**) of the nanoporous silica measured by the Nitrogen (N_2_) Adsorption–Desorption technique using Micromeritics’s TriStar II 3020. The Brunauer–Emmett–Teller (BET) surface area of nanoporous silica was determined to be 722 m^2^/g (**A**) with a pore size of 23 nm using a Density Functional Theory (DFT) model. Scanning electron microscopy images of nanoporous silica particles, consisting of aggregates of several primary particles of the size of 1–10 µm with high porosity (**C**,**D**).

The surface area of the MSP was determined to be 722 m^2^/g via the Brunauer Emmett Teller model with a pore size of 23 nm using a Density Functional Theory (DFT) model. The N_2_ adsorption–desorption isotherm and pore size distribution are shown in Fig. 1A,B. Scanning Electron Microscopy (SEM) was used to characterize the surface morphology and particle size. As shown in Fig. 1C,D, the particles consist of spherical particles in the size range of 1–10 microns, which aggregate to form larger particles in the 10s of micron size range.

### Implantation of MSPs loaded with mimetics, empty particles, and bNCSCs delayed disease onset and extend survival of mutant SOD1^G93A^ mice

First, Cintrofin, Gliafin, and Vefin1 mimetics were loaded into MSPs using the following procedure. MSPs were pretreated in a vacuum oven at 120 °C for 3 h before use. The peptide was dissolved in aqueous solution, and a given amount of pretreated particles were added to the peptide solution and stirred at 4 °C for one day. The mixture was then freeze-dried until most of the water was removed through successive freezing and thawing under vacuum. The loading amount was targeted to be 8–10%, according to previously published protocol^34^. Due to the different water content in the final samples, the final loading amount was calculated by the weight loss between 100 and 900 °C.

Before starting the *in-vivo* experiments, we verified the neurotrophic effect of Vefin1 on MNs in vitro (Supplementary Fig. S1). Immediately after plating, mESC-derived MNs were stimulated with serially diluted Vefin1 peptide for 24 h, followed by fixation with 4% formaldehyde and stained with Hoechst 33342. Vefin1 dose-dependently promoted neurite outgrowth from wildtype MNs, whereas the effect on SOD1^G93A^ MNs was not significant (Supplementary Fig. S1). The neurotrophic effect of Vefin1 on MNs in vitro suggests that application of Vefin1 before disease onset could affect MNs and delay their degeneration. Interestingly, SOD1^G93A^ MNs in vitro did not show a neurotrophic response to Vefin1 (Supplementary Fig. S1). This renders it unlikely that the observed improvements in peptide injected animals were caused by Vefin1 directed response onto MNs.

**Figure 2:**
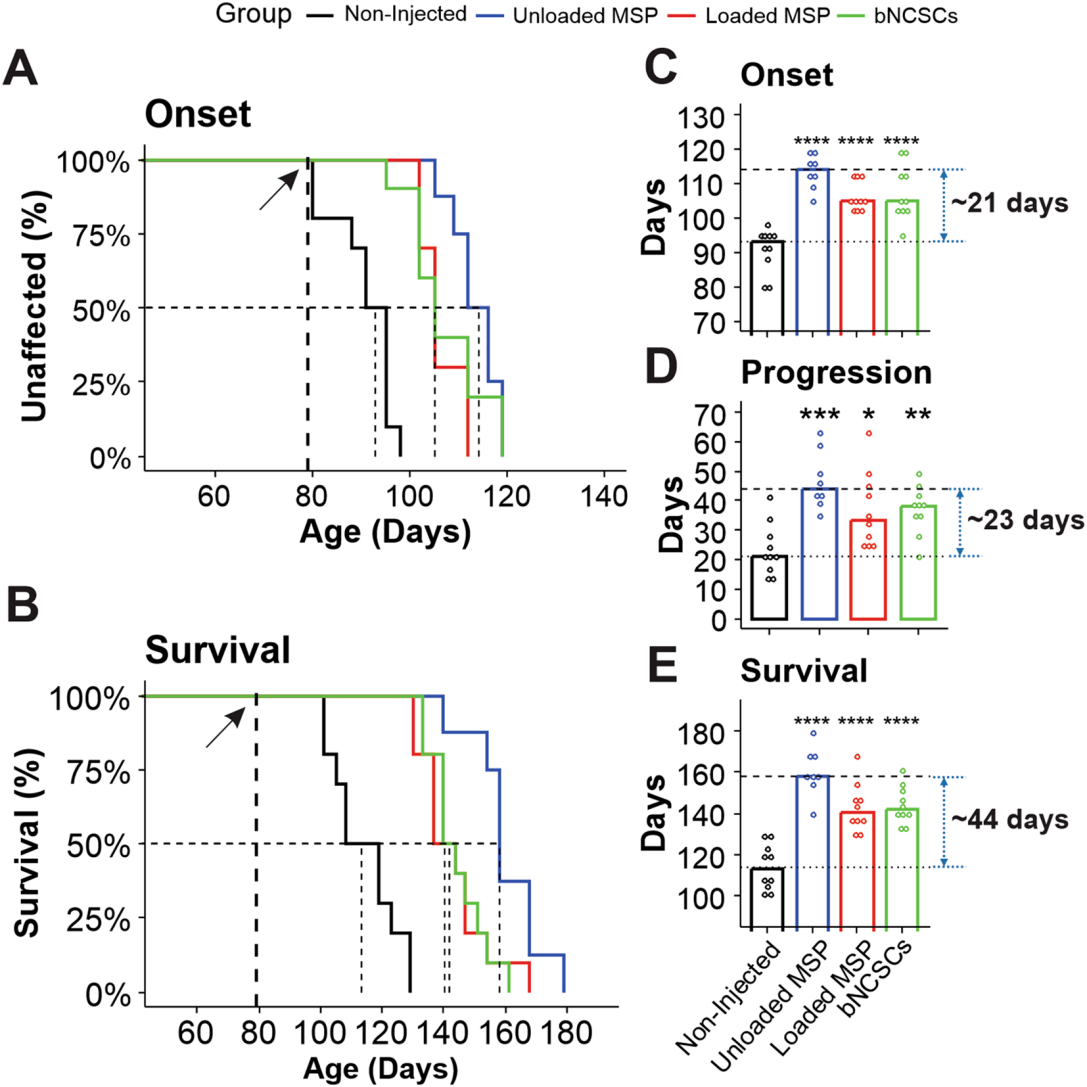
Implantation of empty MSPs, bNCSCs, or MSPs loaded with mimetics into the spinal cord delays disease onset and extends survival of mutant SOD1^G93A^ mice. SOD1^G93A^ female mice received a single intraspinal injection of empty MSPs (n = 8, blue), MSPs loaded with mimetics (n = 10, red), or bNCSCs (n = 10, green) at P79. Treated and untreated (n = 10, black) mice were monitored up to end-stage. MSP or bNCSCs injection into P79 SOD1^G93A^ mice significantly delayed median disease onset (**A**,**C**) compared to non-injected mice (control: 90 days; MSPs-mimetics: 106 days; bNCSCs: 107 days; empty MSPs: 114 days, *p* < 0.001) and extended median disease progression (control: 24 days; MSPs-mimetics: 37 days; bNCSCs: 37 days; empty MSPs: 44 days, *p* < 0.001) (**D**) and median survival (**B**,**E**) (control: 114 days; MSPs-mimetics: 140 days; bNCSCs: 142 days; empty MSPs: 158 days, *p* < 0.001). Mean age of disease onset was determined as the time when mice reached peak body weight. Disease progression was defined as the time from onset (peak body weight) to end-stage. Disease end-stage was determined as the time when the mouse could not right itself within 20 s when placed on its side. At each time point, *p* value was determined by one-way ANOVA with Tukey posthoc analysis. Dot-plot represents the distribution of the individual cases (*p < 0.05).

For survival experiments, SOD1^G93A^ mice injected with mimetic-loaded nanoparticles or boundary cap neural stem cells (bNCSCs) were always compared with littermates injected with empty nanoparticles or non-injected mice. We examined how injection of MSPs unloaded or loaded with mimetics or bNCSCs into the spinal cord affected the disease course in mutant SOD1 mice using the B6SJL-TgN-SOD1-G93A-1Gur (SOD1^G93A^) mice heterozygous for the mutant SOD1 transgene. These mice developed progressive motor neuron disease with symptoms observed from day 90^4^. Seventy-nine-day-old female mice (a time point near disease onset, just before the initiation of weight loss) were injected with nanoparticles containing mimetics (n = 10), empty nanoparticles (n = 8), or bNCSCs (n = 10) (Fig. 2).

Surprisingly, while disease onset (measured by weight loss due to denervation-induced muscle atrophy (Supplementary Fig. S2) was significantly delayed by a median of 13 days for SOD1^G93A^ mice injected with MSP-containing mimetics and by 14 days for SOD1^G93A^ mice injected with bNCSCs compared to SOD1^G93A^ non-injected littermates, the implantation of empty MSPs delayed disease onset by a median of 21 days (non-injected: 93 days; MSP-mimetics:106 days; bNCSCs: 107 days; empty MSPs: 114 days; *p* < 0.001) (Fig. 2A,C). Furthermore, survival of SOD1^G93A^ mice was significantly extended by implantation of MSPs loaded with mimetics or bNCSCs, with the strongest effect obtained with empty MSPs, yielding median survival time of 26 days, 28 days and 44 days, respectively, beyond that of the SOD1^G93A^ non-injected littermates (non-injected: 114 days; MSP-mimetics: 140 days; bNCSCs: 142 days; empty MSPs: 158 days; *p* < 0.001) (Fig. 2B, E).

Defining disease duration as the time from onset to end-stage revealed that the nanoparticle-treated groups had significantly increased duration, indicative of slower disease progression, compared with the SOD1^G93A^ littermates (non-injected: 21 days; MSP-mimetics: 34 days; bNCSCs: 35 days; empty MSPs: 44 days; *p* < 0.001) (Fig. 2D).

### Implantation of unloaded MSPs, MSPs loaded with mimetics, and bNCSCs attenuates the decline of muscle function in mutant SOD1^G93A^ mice

**Figure 3:**
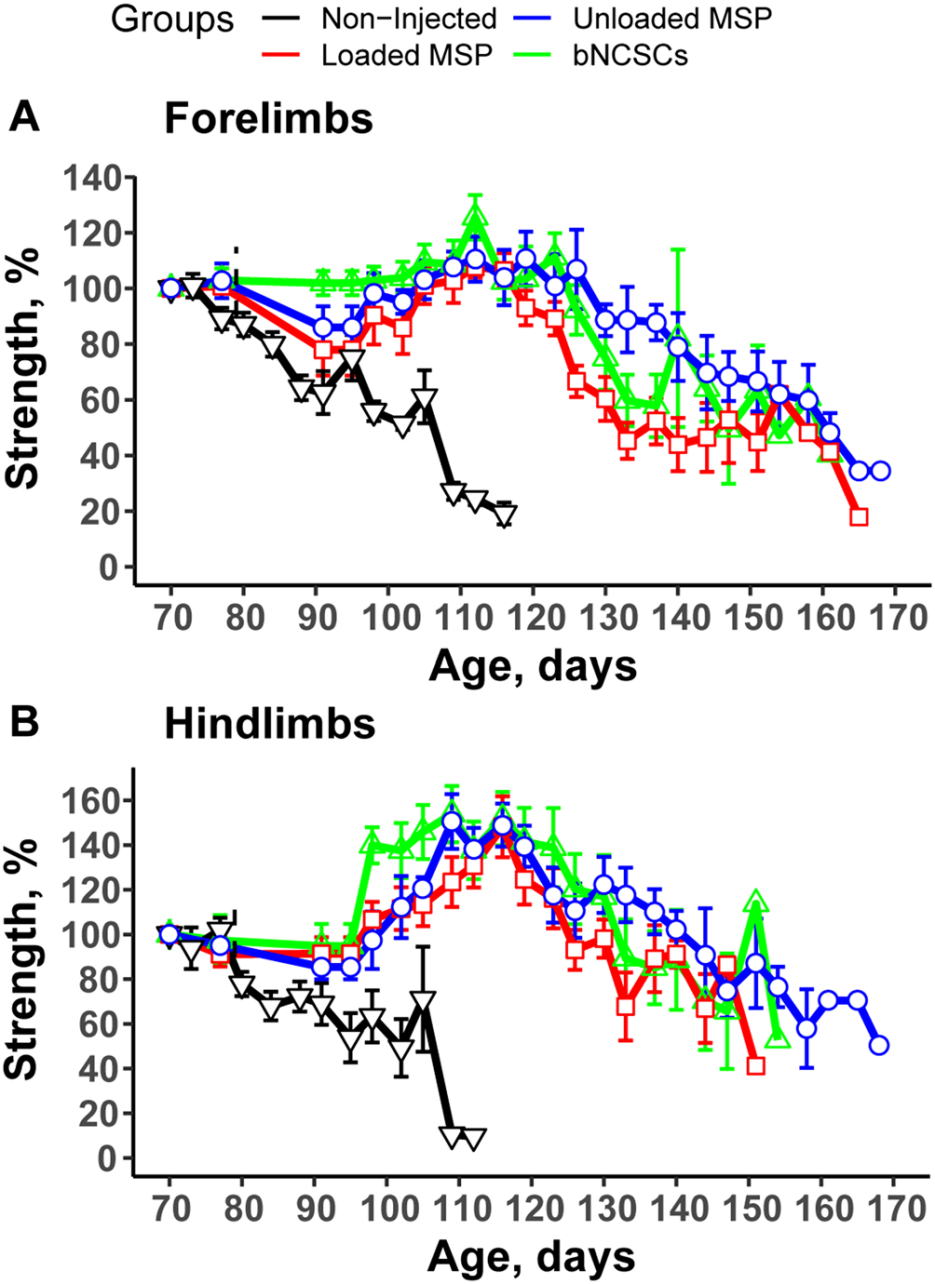
MSP-treated SOD1^G93A^ mice had better forelimb and hind limb grip strength compared with age-matched SOD1^G93A^ mice. Plot of averaged forelimb (**A**) and hind limb (**B**) grip strength percent for SOD1^G93A^ mice uninjected (n = 10, black), injected with MSPs loaded with mimetics (n = 10, red), injected with bNCSCs (n = 10, green), or injected with empty MSPs (n = 8, blue) at indicated age. Data was collected until the limbs became spastic. Error bars denote SEM.

Examination of hind limb and forelimb grip strength showed a rapid decline in non-injected mice after disease onset (Fig. 3). The decline in groups subjected to injection of loaded MSPs or bNCSCs was delayed and followed a similar time course. However, animals subjected to injection of empty MSPs maintained their limb muscle strength for a significantly more extended period of time. Overall, the empty MSP-treated group showed a significantly (*p* ≤ 0.01, multi t-test) greater pulling strength from forelimbs (Fig. 3A) and hind limbs (Fig. 3B) during disease progression compared to the groups receiving mimetics or bNCSCs. These results suggest that mice injected with empty MSPs retain their muscular strength properties during prolonged survival.

### MSP distribution and associated glial response following intraspinal injection

**Figure 4:**
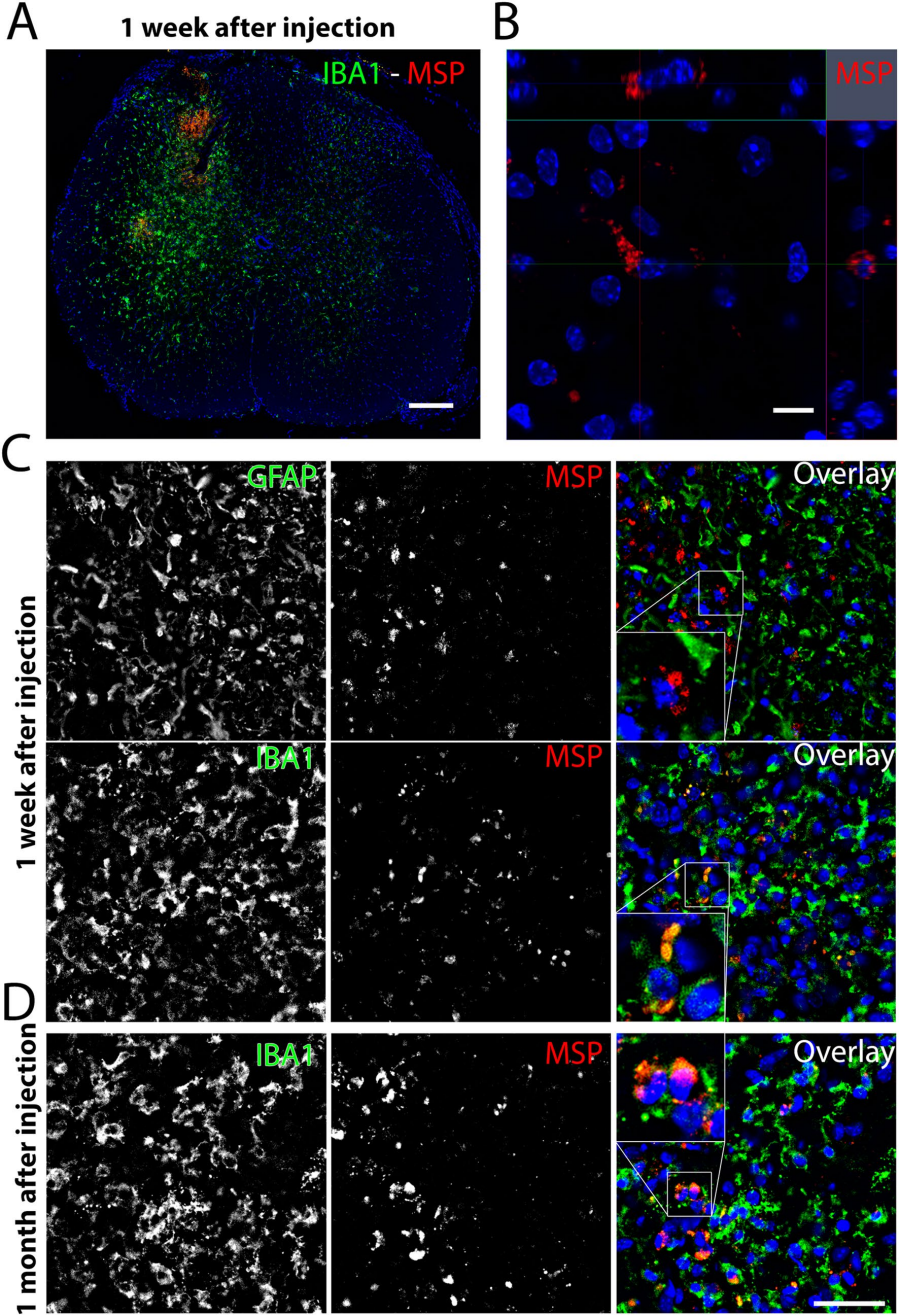
Distribution of Rh-MSP (red) injected into the cervical spinal cord. 1 week after injection Rh-MSP (red) were found in the dorsal and ventral horn associated with a pronounced increase in IBA1^+^ (green) immunoreactivity (**A**) in direct vicinity to DAPI^+^ nuclei (blue) (**B**). One week after injection, Rh-MSP injected areas showed a strong IBA1^+^ microglia (green) and GFAP^+^ astrocytic (green) response, while Rh-MSP (red) primarily associated with microglia (**C**). One month after injection, Rh-MSP (red) could still be found in the cervical spinal cord and remained associated with IBA1^+^ microglia (green). Scale bar = 100 µm (**A**); 10 µm (**B**); 40 µm (**C**).

MSP injections into the spinal cord of SOD1^G93A^ mice were performed with unlabeled MSP. To determine how these particles are distributed after injection we injected Rhodamine labelled particles (Rh-MSP) into the C3–C5 spinal cord segments of wild type mice. We detected Rh-MSP in the dorsal and ventral horn of the spinal cord one week postoperatively ipsilateral to the injection (Fig. 4A). MSP were primarily localized in direct vicinity of DAPI^+^ nuclei (Fig. 4B). At one week, an astrocyte and microglial reaction was seen on the injection side along the needle track, where MSP co-localized with the microglia marker IBA1 but not with the astrocytic marker GFAP (Fig. 4C). One month after injection, particles were still present in the dorsal and ventral horn of the spinal cord and located in direct vicinity of IBA1 positive microglia (Fig. 4D).

## Discussion

The goal of our project was to test a new in vivo approach to deliver growth factor peptide mimetics or stem cells to the ALS-diseased spinal cord to delay disease progression. We show that cervical intraspinal injection of mimetic-loaded mesoporous silica particles (MSPs) or bNCSCs resulted in a delay in disease onset, progression, and lethality compared to non-injected mutant SOD1 mice.

Several neurotrophic factors are known to promote MN survival and differentiation, and have therefore been considered for possible treatment of ALS. Among them, GDNF, CNTF, and VEGF have prominent trophic and survival-promoting effects in models of MN degeneration both in vitro and in vivo^35–39^. These neurotrophic factors work in picomolar ranges and across species, presumably due to being evolutionary conserved molecules. However, despite promising results in pre-clinical studies, clinical trials based on direct administration of these proteins, including CNTF and VEGF, have failed, partly due to ineffective route of administration (i.e., systemic vs. more local), limited ability of such relatively large proteins to cross the blood–brain barrier, and increasing evidence that multiple trophic factors are needed to mitigate the degenerative process^18^. Indeed, recent pre-clinical studies have suggested that the combination of several growth factors is a promising approach to delay the progression of ALS and increase axonal survival and motor performance^18,40^. The combined release of GDNF, VEGF, and NCAM from implanted gene-modified mesenchymal stem cells was beneficial in a mouse model of ALS^36^. In this study, we therefore chose to use a cocktail of Cintrofin, Gliafin, and Vefin1 to achieve a broad coverage of neurotrophic and pro-survival actions.

Short GDNF- and CNTF-derived peptides, Gliafin and Cintrofin, respectively, have previously shown robust neurotrophic and pro-survival effects in vitro^41,42^. In our previous studies, we showed that Gliafin and Cintrofin released from loaded MSPs effectively promote MN survival and differentiation in vitro^21^ and in vivo^22^, and therefore were used in this experiment. VEGF has well-known beneficial effects on MNs and survival in a transgenic mouse model of ALS^43^. Vefin1 peptide, the sequence of which is modeled after the binding epitope of VEGF to VEGFR1, has been shown to exert potent neurotrophic and neuroprotective effects in vitro on a number of primary neurons^44^. The neurotrophic effect of Vefin1 on MNs in vitro suggests that application of Vefin1 before disease onset may affect the healthy MNs and delay their degeneration. Thus, including Vefin1 together with Gliafin and Cintrofin represents a novel combinatorial neurotrophic factor-based treatment for early onset of ALS. We have previously shown that peptide mimetics are released over at least a two-week period from loaded MSPs in vivo^22^. To achieve long-term bio-availability of peptide mimetics, we employed the MSP release system for efficient local delivery to the degenerating MNs in an ALS model. This treatment led to significant retardation of muscle weakness, a marked delay in disease onset, and extended survival of the SOD1^G93A^ mutant mice.

Stem cell transplants are treatment options for ALS by serving as a source for neuroprotective and/or neurotrophic factors, as well as for replacement of lost neurons. Numerous experimental studies using different sources of stem cells in addition to different routes of implantation have demonstrated extended survival and modest improvements in motor functions^24,45^. Some of these approaches have been translated into early clinical trials, largely focusing on safety issues and possible side effects from the transplant. The trials show that stem cells can be delivered to the spinal cord at multiple sites without adverse effects, and that stem cell-based therapy holds promise for long-term significant symptomatic improvements^26,27,46,47^. So far, preclinical transplantation of stem cells for ALS has largely focused on their neurotrophic and neuroprotective properties. Here, we tested transplants of bNCSCs, which were previously shown to protect MNs harboring SOD1^G93A^ mutation in vitro and in vivo^33^. This treatment resulted in symptomatic improvement and extended survival, suggesting that bNCSCs were able to, at least in part, withstand the toxic environment created by the mutant, misfolded SOD1. Their positive effects on muscle strength and survival could be a result of their previously demonstrated neurotrophic and angiogenetic capacity^31^. At the end of the experiment, we were able to detect a small number of bNCSCs along meninges, but not in the spinal cord parenchyma (not shown). We previously showed that bNCSCs injected into the spinal cord display strong and directionally diverse migration potential^48^. The absence of bNCSCs within the spinal cord more than two months after their injection could, therefore, be a consequence of their migration from the injection site towards the spinal cord surface.

Unexpectedly, by far the best therapeutic outcome of our experiments was obtained by injection of empty MSPs into the mutant SOD1 mice. Thus, our finding suggests that empty MSPs are able to absorb harmful molecules, attenuate the toxic environmental impact on MNs, and possibly also other neuronal cells in the mutant SOD1 mice, thereby indirectly promoting MN survival and maintaining functional neuromuscular connections. Using primary enriched motor neuron cultures which were subjected to oxidative stress, we have shown that cultures treated with MSP had better survival (Supplementary Fig. S4), supporting our hypothesis.

In the early stage of ALS, symptoms are focal and random, but subsequently progress in a “prion”-like fashion^49^ by extending from focal “nuclei” to neighboring cells, as well as along interconnected neuroanatomical pathways to distantly located neurons. A similar mode of progression has been demonstrated for TAR DNA-binding protein 43 (TDP-43), a pathological protein typically found in cytoplasmic aggregates of MNs in ALS^50^. Misfolded mutant SOD1, as well as misfolded wildtype SOD1, display intercellular transmission, either through uptake by adjacent cells of protein aggregates leaking from dying cells or by endocytosis after release of exosomes containing misfolded/aggregated SOD1 from affected cells^51–53^. Once inside the cell, misfolded SOD1 associates with intracellular organelles as mitochondria and ER^54^ and interacts with specific targets such as VDAC1 in the mitochondria^55,56^ and Derlin1 in the ER^57^ leading to cellular dysfunction and cell death. Thus, empty MSPs by absorbing yet non-identified factor(s) may interfere with intercellular transfer of disease-promoting molecules, which could provide a means to delay or halt disease progression. Alternatively, or additionally, empty MSPs may help to reduce the inflammatory and/or oxidative damage load on endangered MNs^58^, thereby extending their functionality.

The porous structure of MSPs provides a large surface area, allowing for the incorporation of large amounts of drugs within a small carrier and binding to different molecules^59^. Empty MSPs in an extracellular environment are likely to operate in a similar manner and incorporate molecules, which can pass through the silica pores or bind to the silica in a concentration dependent manner. Pore size can be easily modified to match the size of target molecules and the particles further functionalized, e.g., for imaging using fluorescent probes or quantum dots, thus, offering an attractive platform for the development of novel theranostics.

Taken together, MSPs represent a highly innovative molecular scavenging approach to delay the progression of ALS. This approach can be applied by itself or as an additive to improve the environmental conditions for other treatments such as neurotrophic factors or stem cell transplants.

## Materials and methods

### Synthesis of mesoporous silica

Pluronic 123 (triblock co-polymer, EO20PO70EO20, Sigma-Aldrich) (4 g) as a templating agent and, 1,3,5-trimethylbenzene (TMB) (Mesitylene, Sigma-Aldrich) (3.3 g) as swelling agent were dissolved in 127 ml distilled H_2_O and 20 ml hydrochloric acid (HCl, 37%, Sigma-Aldrich) while stirring at room temperature (RT) for 3 days. The solution was preheated to 40 °C before adding 9.14 ml TEOS (Tetraethyl orthosilicate, Sigma-Aldrich). The mixture was stirred for another 10 min at the speed of 500 rpm and kept at 40 °C for 24 h, then hydrothermally treated in the oven at 100 °C for another 24 h. Finally, the mixture was filtered, washed, and dried at room temperature. The product was calcined to remove the surfactant template and swelling agent. The calcination was conducted by heating to 600 °C with a heating rate of 1.5 °C/min and kept at 600 °C for 6 h followed by cooling to ambient conditions. The resulting product was a white powder composed of nanoporous silica particles.

### Physical and chemical characterization of mesoporous silica

Mesoporous silica particles have been characterized previously^60^. The loading amount of peptide was determined by thermogravimetric analyses (PerkinElmer, Waltham, MA, USA). A temperature ramp was performed from 20 °C to 900 °C at a heating rate of 20 °C/min. The plug‐in gas atmosphere was dry air (flow rate, 20 ml/min). The sample weight varied from 5 to 10 mg.

A nitrogen adsorption–desorption isotherm was conducted to characterize the porosity and determine the surface area of the MSP powders (Micromeritics Tristar II 3020 apparatus, Norcross, GA, USA). Calcined MSPs were degassed at 300 °C for 6 h under nitrogen gas flow. Trophic factor-loaded samples were degassed at 60 °C for 48 h prior to analysis. The pore size distribution was calculated using Density Functional Theory (DFT) model, assuming cylindrical pore geometry, using the software package included with the Micromeritics Instrument (Fig. 1)^34^.

Scanning electron microscopy (SEM) images were recorded using a LEO 1550 SEM equipped with a Schottky field emission gun operated at an accelerating voltage of 1–3 kV and 20,000–50,000× magnification on samples without gold coating. Transmission electron microscopy (TEM) of the calcined samples was conducted with a JEOL-3010 (JEOL) microscope operating at 300 kV (Cs, 0.6 mm; 1.7 Å resolution). Images were recorded using a charge-coupled device (CCD) camera (Keen View, SIS Analysis Specialized Imaging, Olympus Soft Imaging Solutions, Münster, Germany; 1024 × 1024 pixels; 23.5 × 23.5 µm field of view) at 30,000–100,000× magnification using low-dose conditions on as-synthesized and calcined samples^34^.

### Functionalization and labeling of mesoporous silica nanoparticles

In order to graft Rhodamine B isothiocyanate on the surface of silica particles, the silica particles were first functionalized by propyl amine groups. Calcined MSP (1 g) were added to toluene (50 ml) and sonicated for 30 min. (3-aminopropyl)-triethoxysilane (APES, Sigma-Aldrich) (650 µl) was dissolved in toluene (5 ml) at room temperature and added to the silica suspension. The mixture was stirred overnight, filtered, washed with ethanol, and air-dried. The resulting aminopropyl functionalized MSPs were named nanoporous silica-NH_2_. Thermogravimetric analysis (TGA) was used to determine the number of aminopropyl groups grafted onto the silica surface. The weight loss of functionalized silica particles in a temperature range of 100–900 °C, which was attributed to the organic propylamine groups, was around 11%.

Rhodamine B isothiocyanate (Rh, Sigma-Aldrich) was used to label the silica particles. Rh (1 mg) was dissolved in methanol (100 ml) and added to 1 g of nanoporous silica-NH_2_. The mixture was stirred at 40 °C for 2 h, filtered, washed with ethanol, and air-dried. The final samples were named Nanoporous silica-Rh.

The surface area and porosity of nanoporous silica-Rh was measured by N_2_ adsorption–desorption (Fig. 1). The surface area of the functionalized silica decreased from 722 to 402 m^2^/g compared to the unfunctionalized silica, while the pore size decreased marginally from 23 to 21.5 nm, which indicated that the porosity of the particles remained available for loading peptide mimetics. The fluorescent property of the labeled particles was visualized by fluorescence microscopy and revealed a very strong fluorescent signal (data not shown).

### Neurotropic factor peptide mimetics

Peptides Gliafin (ETMYDKILKNLSRSR)^41^, Cintrofin (DGGLFEKKLWGLKV)^42^, and Vefin1 (AKFMDVYQRSYSHA)^44^ were synthesized as tetramers composed of four monomers C-terminally coupled to a lysine scaffold using Fmoc solid-phase peptide synthesis (Schafer-N, Copenhagen, Denmark). The peptide purity was ≥ 95%, as determined by high-performance liquid chromatography.

### Loading mesoporous silica particles with peptide mimetics

Cintrofin, Gliafin, and Vefin1 were loaded in mesopores via impregnation in water as previously described^34^. Briefly, MSPs were added to aqueous solutions of each peptide and stirred at 4 °C for 16 h, followed by water evaporation under atmospheric conditions. The loading amount of peptides was determined by thermogravimetric analysis (PerkinElmer, Waltham, MA, USA) by scanning samples from 20 to 900 °C at a heating rate of 20 °C/min. The plug-in gas atmosphere was dry air with flow rate 20 mL/min. Loading amounts of Cintrofin, Gliafin, and Vefin1 were 8.3 wt%, 11.8 wt%, and 8 wt%, respectively. Accordingly, the adsorption isotherm data showed a considerable reduction in the pore volume and pore size of the MSPs after the incorporation of mimetics. Powder X-ray diffractograms of loaded samples revealed that the peptides were loaded in an amorphous state, as diffraction peaks were not observed. The release kinetics of peptide-loaded mesoporous silica particles was demonstrated previously^34^.

### Culture of mouse embryonic stem cell (mESC)-derived motor neurons for in vitro testing of Vefin1 neurotrophic activity

Since the neurotrophic efficacy of the peptide mimetic Vefin1 on MNs was not documented previously, we set up the same in vitro system as was previously used for Cintrofin and Gliafin^21^. A mouse ESC line carrying GFP-encoded genes under the control of the MN specific transcription factor HB9 (HB9:GFP) (gift from Dr. Kevin Eggan, Harvard Stem Cell Institute) was directly differentiated in vitro as described previously^61^. Three independent experiments were conducted. Briefly, mESCs were propagated on top of mouse embryonic fibroblasts in ES (Embryonic Stem) cell culture medium containing 82% KoDMEM with high glucose, 1% GlutaMAX, 1% Penicillin–Streptomycin, 1% Non-Essential Amino Acids (all from Life Technologies, Carlsbad, USA), 14% KnockOut Serum Replacement (KoSR, Invitrogen, Eugene, USA), 100 µM beta-Mercaptoethanol (ME), and 1% ESGRO Leukemia Inhibitory Factor (LIF, Millipore, Billerica, USA). To initiate neuronal differentiation, dissociated mESCs were re-suspended in ADFNB medium containing Advanced DMEM/F12:Neurobasal (1:1 v/v), 0.5% N2, 1% B27 (all from Life Technologies), 1% GlutaMAX, 1% Penicillin–Streptomycin, 1% KoSR, and 100 µM beta-ME. Two days later, when cells formed uniform embryoid bodies (EBs), media was exchanged to fresh ADFNB supplemented with 0.1 µM retinoic acid (Sigma, St. Louis, USA) and 0.2 µM Shh agonist Ag1.3 (Phoenix Pharmaceuticals; Burlingame, USA), and cells were cultured for five consecutive days with media exchanges every other day. For MN cultures, EBs were dissociated and plated at a density of 7.5 × 10^4^ cells/coverslip on 0.01% poly-l-ornithine and 10% Laminin (Sigma) pre-coated glass cover slips in ADFNB medium. Immediately after plating, cells were stimulated with serially diluted Vefin1 peptide for 24 h. Treated and untreated cultures were then fixed with 4% formaldehyde and stained with 1:10,000 Hoechst 33342 (Invitrogen, Eugene, USA). Neurite outgrowth from GFP-expressing MNs was analyzed as previously described^44^.

### Motor neuron culture

Primary motor neuron cultures were prepared as previously described^62^. Motor neurons were isolated and plated in eight-well LabTek Permanox chamber slides (Nunc, Roskilde, Denmark) pre-coated with poly-L-Ornithine and laminin. Cells were cultured for 7–10 days in Neurobasal medium supplemented with 2% B27, 2% (v/v) horse serum, glutamine 100 U/ml penicillin, and 100 μg/ml streptomycin. To induce cellular death, cells were treated with H_2_O_2_ (60 µM; Sigma-Aldrich) alone or together with 10 µl of empty MSPs for 48 h. For each individual experiment, a well with unstimulated cells were used as a positive control. Supernatant was collected, centrifuged at 12.000×*g*, and the level of glutamate was estimated by Glutamate Assay Kit (Sigma). Cells were fixed with 4% formaldehyde and nuclear morphology was visualized with Hoechst 33258 (1:1000, Invitrogen). Random images were recorded using Zeiss Axiovert 100 microscope mounted with an AxioCam MRm camera (Zeiss) and cellular survival was assessed as previously described^44^.

### Culture of boundary cap neural crest stem cells (bNCSCs)

bNCSCs were generated from transgenic mice harboring red fluorescent protein (RFP) under the universal actin promoter^63^ according to previously published protocols^64,65^. Briefly, the DRGs along with boundary caps were mechanically separated from the isolated spinal cord and mechano-enzymatically dissociated using Collagenase/Dispase (1 mg/ml) and DNase (0.5 mg/ml) for 30 min at room temperature. Cells were plated at 0.5–1 × 10^5^ cells/cm^2^ in an N2 medium containing B27 (Gibco) supplemented with EGF and bFGF (each 20 ng/ml; R&D Systems (Abingdon, United Kingdom). After 12 h, non-adherent cells were removed together with half of the medium, which was replaced by fresh medium. The medium was changed every other day (50% of the medium replaced with fresh medium) until neurospheres were formed (approximately two weeks of culture). Non-passaged neurospheres between two and three weeks in culture were used in subsequent experiments.

### Animals

Altogether 40 B6/SJL background (B6SJL-TgN-SOD1-G93A-1Gur; SOD1^G93A^) female mice were obtained from Jackson Laboratory (Maine, US), and 6 NMRI mice from Möllegaard and Bomholgard Breeding and Research Centre (M&B A/S, Bomholt, Denmark,) were used for the experiments. All experiments were carried out in accordance with the European Union Directives and Society for Neuroscience Guidelines for research on animals. All efforts were taken to minimize animal suffering. For B6SJL-TgN-SOD1-G93A-1Gur animals, the treatment protocol was approved by the Animal Care and Use Committee of Ben-Gurion University of the Negev, as required by Israeli legislation, and for the NMRI animals, approval was given by the Uppsala Ethics Committee for Research on Animals.

### Surgery

Mice were anesthetized prior to surgery with 3% isoflurane at the beginning of the procedure, and maintained over time with 0.8% at a flow rate of 500–480 ml/min isoflurane. The skin on the dorsal part of the neck was incised, and the left C3–4 vertebral laminae exposed and removed to expose the left dorsal part of the corresponding spinal cord segments (Supplementary Fig. S3A, B).Three injections (2 µl each) were made into the ventral part on the left side of the cervical spinal cord (C3–C4) with a 10-µL Hamilton syringe attached to a micro syringe pump controller (Micro 4, World Precision Instruments, Sarasota, FL) (Supplementary Fig. S3C). SOD1^G93A^ mice were operated on at 79 days of age (~ 10 weeks) and divided in three experimental groups: (1) control group (n = 10; unloaded (empty) MSPs), (2) peptide mimetic group (n = 10; Cintrofin, Gliafin, and Vefin1 loaded MSPs), and (3) bNCSC group (n = 10; ~ 26,000 cells per injection). NMRI mice (n = 6) were operated on at a similar age and in the same way with an injection of 2 µL Rhodamine-labeled mesoporous silica nanoparticles (Rh-MSPs). Mice recovered without complications after surgery (Supplementary Fig. S3D).

### Immunohistochemistry

NMRI mice were re-anesthetized one week (n = 3) and one month (n = 3) after surgery, and perfused with warm saline (~ 38 °C), followed by a fixative solution containing ice-cold 4% w/v formaldehyde in phosphate-buffered saline (PBS; ~ 4 °C, pH 7.35–7.45). The C3–5 spinal cord segments were removed, post-fixed at 4 °C for 4 h, and cryoprotected overnight in PBS containing 15% sucrose. Serial coronal sections (14 μm) were cut through the spinal cord on a cryostat, and placed on SuperFrost Plus glass slides (Menzel-Gläser, Braunschweig, Germany). Sections were preincubated with blocking solution (1% bovine serum albumin, 0.3% Triton X-100, and 0.1% NaN3 in PBS) for 1 h at room temperature and then incubated overnight at 4 °C with primary antibodies to glial fibrillary acidic protein (GFAP; rabbit polyclonal, 1:500, Dako) or IBA1 antibodies (microglial marker; goat polyclonal., 1:500, Abcam). Immunohistochemistry was performed as described previously^23,33^. After washing with PBS, appropriate secondary antibodies were applied for 4 h at room temperature, and cell nuclei were labeled with Hoechst. Sections were examined using a LSM700 confocal microscope (Zeiss).

### Behavioral assessment

After surgery, mice were weighed daily throughout the entire observation period, and tested for grip strength twice a week as described previously^66^, beginning two weeks before surgery. Time of disease onset was retrospectively determined as the time when mice reached peak body weight. Early disease was defined at the time when denervation-induced muscle atrophy had produced a 10% loss of maximal weight. End-stage was determined by paralysis so severe that the animal could not right itself within 20 s when placed on its side, an endpoint frequently used for SOD1 mutant mice and one that is consistent with the requirements of the Animal Care and Use Committee of Ben-Gurion University of the Negev. Grip strength testing was performed with a Chatillon force measurement device (Ametek). Measurement of each integrant of the groups consisted of two measurements (forelimb pulling and total limb pulling).

### Statistical analysis

Data were collected throughout the study and analyzed using RStudio Team (2020) version 1.3.959. (RStudio: Integrated Development for R. RStudio, PBC, Boston, MA URL http://www.rstudio.com/). Kaplan Meier curves were generated with *survival* package v3.2-3 and plotted with *survminer* package v0.4.7. Bar and line plots were generated with *ggplot2* package v3.3.2. Statistical analysis was further performed with *stats* v3.6.3 (R Core Team) and other packages loaded into the RStudio environment, *dplyr* v1.0.0, *tidyverse* v1.3.0., and *multicomp* v1.4–13. Multicomparison of the means was done using one-way ANOVA with Tukey posthoc analysis, being a *p* value of < 0.05 statistically significant (****< 0.001, ***< 0.001;**< 0.01; *< 0.05) loaded in the *multicomp* RStudio.

## Supplementary information

Supplementary Information.
