# Supplementary material for: Empty mesoporous silica particles significantly delay disease progression and extend survival in a mouse model of ALS
Source: Sci Rep. 2020 Nov 26;10:20675. doi: 10.1038/s41598-020-77578-x (PMC7691331; doi:10.1038/s41598-020-77578-x)
Supplement: Supplementary file 1 — Supplementary Information. [file 41598_2020_77578_MOESM1_ESM.pdf]

Supplementary information

Empty Mesoporous Silica Particles Significantly Delay Disease Progression and Extend Survival in a Mouse Model of ALS

Marcel Leyton-Jaimes[1#], Patrik Ivert[2#], Yilin Han[2&], Jan Hoeber[2,7&], Adam Feiler[3,4], Chunfang Zhou[4], Stanislava Pankratova[5], Varda Shoshan-Barmatz[6], Adrian Israelson[1*], Elena N Kozlova[2*]

Supplementary Figures

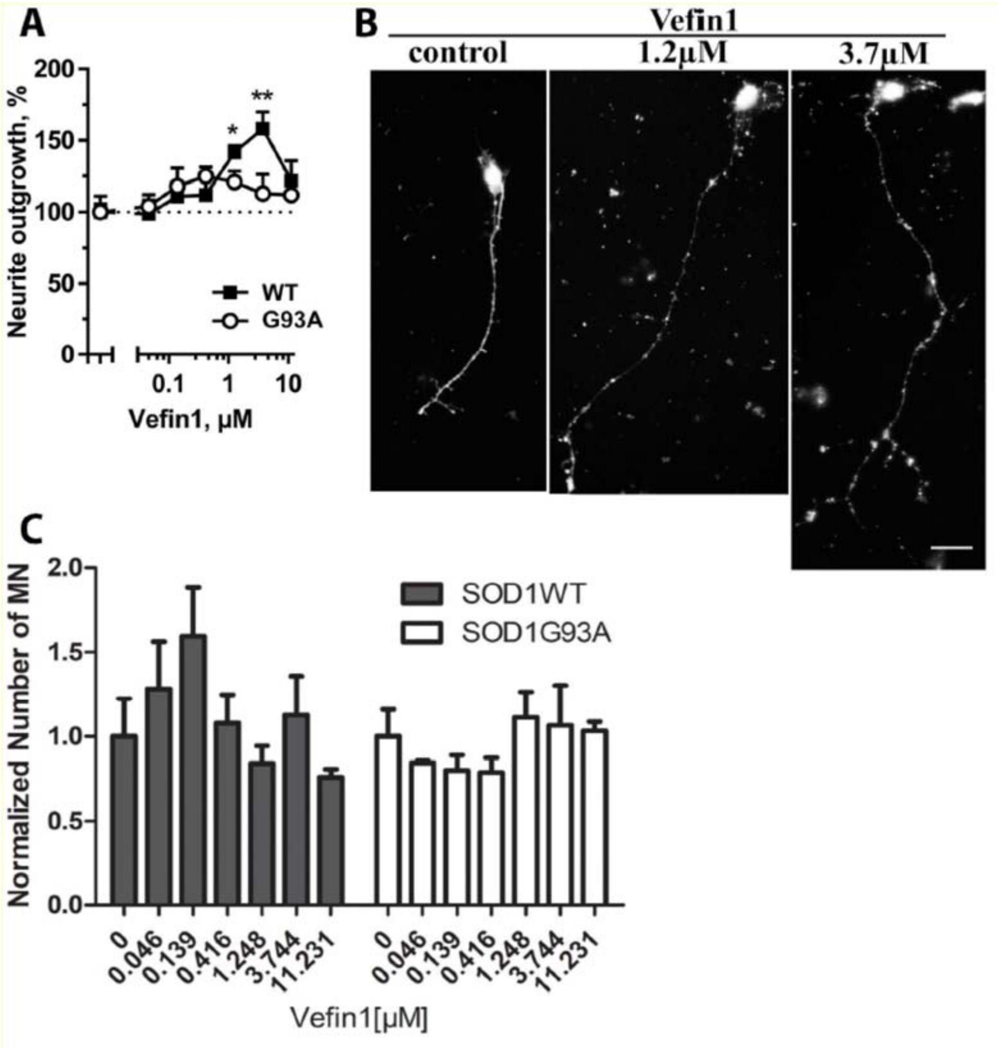

Fig. S1: Neurotrophic effect of Vefin1 peptide on cultured mouse ESC-derived motor neurons. (**A**-**C**) Differentiated motor neurons expressing GFP fluorescent protein were stimulated with serially diluted Vefin1 peptide for 24 h. Vefin1 dose-dependently promotes neurite outgrowth from wildtype but not from G93A-mutated neurons. Results from three independent experiments expressed as means ± SEM, with untreated control set to 100% (A, dashed line). *$p < 0.05$, **$p < 0.01$, compared to untreated control. The neurite outgrowth shown in **B**. Scale bar 25µm. The quantification of normalized number of motor neurons is shown in **C.**

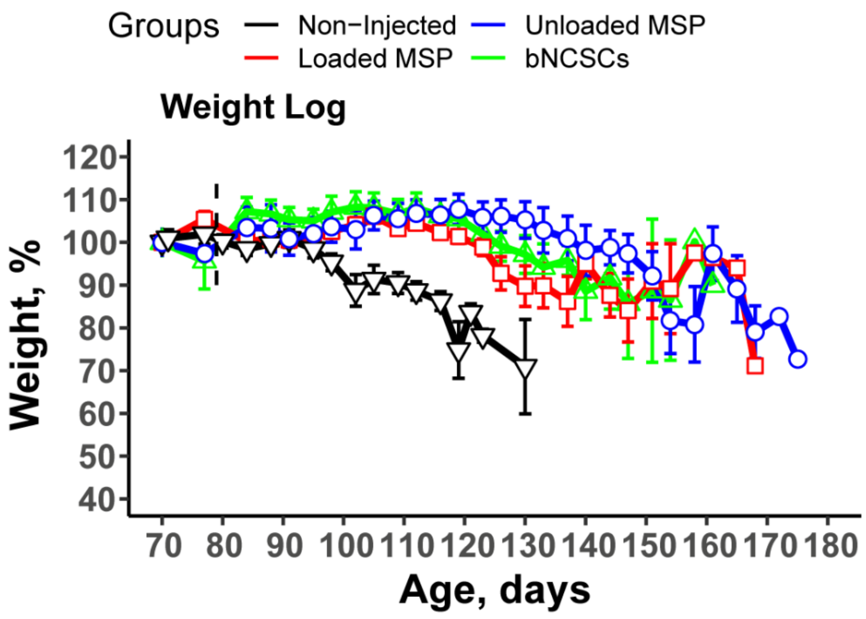

Fig. S2: MSP-treated SOD1^{G93A} mice sustained better weight compared with age-matched SOD1^{G93A} mice. Plot of averaged percent of weight for SOD1^{G93A} female mice uninjected (n=10, black), injected with MSPs loaded with mimetics (n=10, red), injected with bNCSC cells (n=10, green), or injected with empty MSPs (n=8, blue) at indicated age. Error bars denote SEM.

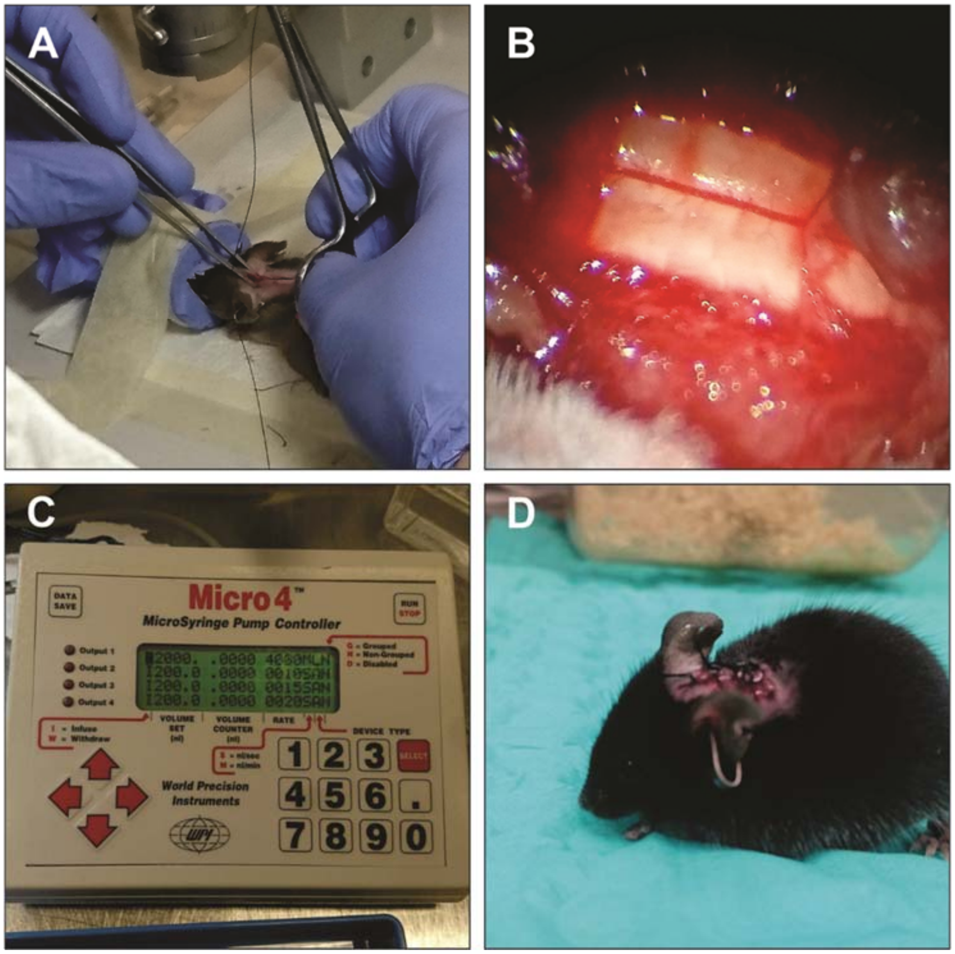

Fig. S3: Mice surgery overview. (**A**) Anesthetized mice being stitched after the procedure. (**B**) 20X magnification of the spinal cord exposed before injection. (**C**) Microsyringe pump device which allowed controlled and precise injection to the mice spinal cord. (**D**) Mice recovering in a water-based controlled warming pad after surgery.

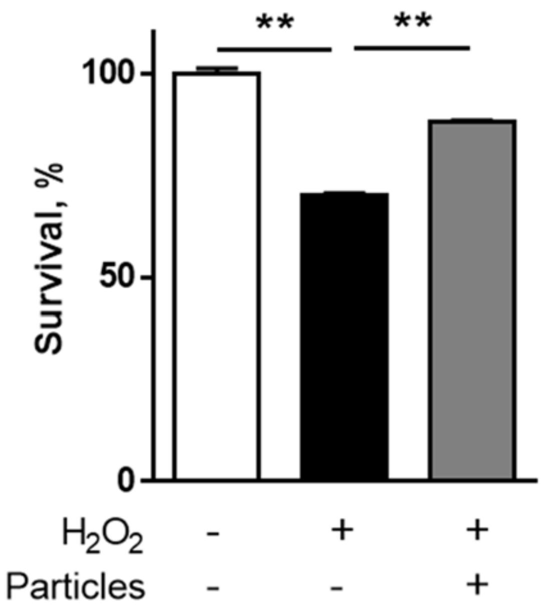

Fig. S4. Effect of empty MSP on survival of cultured motor neurons challenged with oxidative stress. Primary motor neurons isolated from E14 spinal cord were treated with H_2O_2 alone or in combination with MSP for 48 hours. Untreated cultures were used as baseline control. MSP treatment significantly improves motor neuron survival compared to untreated cultures ($p < 0.01$).
